# Emergency management for severe burn (EMSB) course for the nurses in Bangladesh: opportunity and way forward

**DOI:** 10.1016/j.heliyon.2022.e09156

**Published:** 2022-03-23

**Authors:** Animesh Biswas, Koustuv Dalal, Rifat Ara Sawon, Cinderella Akbar Mayaboti, Saidur Rahman Mashreky

**Affiliations:** a Centre for Injury Prevention and Research, Dhaka, Bangladesh; b Department of Epidemiology, Biostatistics and EBM, Faculty of Medicine and Health Care, al-Farabi Kazakh National University, Almaty, Kazakhstan; c Division of Public Health Sciences, School of Health Sciences, Mid Sweden University, Sundsvall, Sweden

**Keywords:** Burn, Emergency, Nursing, Training, Bangladesh

## Abstract

**Background:**

The emergency management of severe burn (EMSB) course is one of the widely taken courses in over 15 courses worldwide. In Bangladesh, the course has been running since 2008. Over 600 doctors and only 72 nurses participated in the EMSB courses in Bangladesh. The study explored the experiences of the EMSB course for the nurse, including opportunity and way forward.

**Methodology:**

A multi-method study was conducted. Quantitative data were collected from 54 nurses using the telephone interviews. In addition, one focus group discussion was performed with the EMSB faculty members to obtain qualitative information.

**Results:**

Out of 54 participant nurses, 47(87.04%) were female, and 7 (12.96%) were male. Almost two-thirds of nurses (62.96%) were working at medical colleges and hospitals. About 52% of the respondents stated that they had the opportunity to use the knowledge and skill acquired from EMSB training in managing burn patients. Those who had a chance to use the EMSB course knowledge, among them a vast majority (92.8%) mentioned that it helped manage severe burn patients. However, every nurse struggled with the course language. As a result, they were not able to qualify for the written course examination. They were also not able to interact well during the lecture sessions. However, nurses did well in the moulage practical simulation session.

**Conclusions:**

Immediate management of burn at the facility level could reduce disease burden, including hospital stay and quality of life. Nurses EMSB course, therefore, is essential for burn management in Bangladesh. Furthermore, course content updating, including bilingual option, could improve the nurse's course completion rate and confidence to contribute to their job areas.

## 1 Introduction

In terms of morbidity and disability, burn is a significant public health problem throughout the world, especially in developing countries [1]. The cost of burn treatment further compounds the problem. It requires specialized personnel and technologies that are not always readily available in many of the low-income countries [2, 3, 4, 5, 6, 7]. Every year in Bangladesh, more than 365,000 people are injured by electrical, thermal and other causes of burn injuries. Among them, 27,000 needed hospital admission, and over 5,600 died [8]. Children are the most vulnerable group for burn and electrical injury. Approximately 173,000 children suffer from thermal injuries, and about 45,000 children suffer from electrical injuries every year in Bangladesh. Burns was found as the 5th leading cause of illness among 1–17 years old children and the 3rd leading cause of illness among children of 1–4 years [9, 10]. Burns are a major cause of school absence and workday loss for children in Bangladesh, and about 3,400 children become permanently disabled due to burn-related injury [10]. A vast majority of hospital admission and hospital stay is due to burn injury [10], causing a substantial economic and social burden for families [10, 11]. Rural children are more vulnerable to burn injuries compared to urban [11].

Considering the magnitude of burn injury, Australia New Zealand Burn Association (ANZBA) has come forward to support Bangladesh physicians to improve burn management. ANZBA initiated EMSB training program for Bangladesh physicians in 2008. Interplast Australia New Zealand provided financial support to conduct this training in Bangladesh. Centre for Injury Prevention and Research Bangladesh (CIPRB) organized this EMSB program in Bangladesh with ANZBA and the burn professional of Bangladesh. The course is one day long, consisting of four parts, lectures, practical sessions, interactive discussions and a test composed of theory and practice [12]. Between 2008 and 2017, about 600 burn physicians and another 72 nurses of Bangladesh received EMSB training under this project.

Moreover, around 20 doctors and nurses from Nepal also received the course. Forty-one physicians have been selected and trained as EMSB instructors. A short evaluation was performed in 2013 to evaluate doctor's courses; however, the effects of EMSB courses for the nurses have not been examined. Therefore, this study has explored the effects of the EMSB course for nurses in Bangladesh.

## 2 Methods

### 2.1 Study design

A multi-method study was performed where both quantitative and qualitative data were collected. A cross-sectional survey was conducted among the nurses who received EMSB training in Bangladesh for obtaining information about the utilization of knowledge and skill of the EMSB course. A cellphone-based data collection technique was used, a pre-tested structured questionnaire was utilized to obtain quantitative data. In addition, one focus group discussion (FGD) was performed with the EMSB faculty members to get in-depth qualitative information. 12 EMSB faculty members have participated in the FGD. Out of them, three were key instructors, eight were faculty members from Bangladesh and EMSB course director of Australia, and the New Zealand Burns Association (ANZBA) participated. Consolidated Criteria for Reporting Qualitative Studies (COREQ) checklist for reporting the focus group discussion was followed and appended [13]. Verbal consent was taken before conducting the interview and FGD. Objectives of the study and utilization of the data were mentioned to each of the participants.

### 2.2 Sampling for survey

A total of 72 nurses completed the EMSB course successfully and out of these 72 nurses, we were able to locate 54 participants in the evaluation.

### 2.3 Data collection procedure

EMSB database was used to obtain the contact details of participants. Trained professionals interviewed the nurses through a phone call. Verbal consent was ensured before the interview conduction, and on average, the interview lasted for 20 min. The interviewer filled out a hard copy of questionnaire. If the participant did not receive the call the first time, the next two days, we tried to reach them. Among 72 nurses, 54 participated in this study.

For qualitative data, the study's principal investigator, who is the key coordinator of EMSB in Bangladesh, facilitated the FGD using an unstructured guideline. The audio was recorded using a voice recorder before the permission of the respondents. Keynotes were also taken.

### 2.4 Data analysis

Quantitative data was analyzed using SPSS for windows version 22. Descriptive analysis, like proportion frequency, was done. Content analysis was performed for qualitative data.

### 2.5 Ethical issue

This study was not an experimental or not designed to collected patient's individual data. According to rules, ethical approval was not mandatory. However, the whole project has received ethical permission from the Institutional review board of the Centre for Injury Prevention and Research, Bangladesh. Written informed consent was received from all participants participated in the telephone interviews. Verbal consent was taken from the participants before the FGDs.

## 3 Results

Total of 54 nurses participated in this study; among them, 47(87.04%) were female, and seven (12.96%) were male. Among them, 37.04% completed diploma course, 50.0% completed bachelor of Science (BSc) and rest completed some other courses, including SSC, HSC, Midwifery, and Junior nursing course. More than half of the respondents (62.96%) were working at medical colleges and hospitals. When segregated department wise, the majority (42.6%) were working at surgery department, 24.1% were burn and plastic department, rest were from the medicine department, intensive care/emergency unit and nursing supervisor/instructor/matron (Table 1).

**Table 1:** Overview of the respondents participating in the study.

Overall nurses status	Frequency	Percentage (%)
**Gender**		
Male	11	13.0
Female	74	87.0
**Education**		
Diploma	32	37.0
BSc	43	50.0
Others	10	13.0
**Workplace**		
Medical college or Specialized Hospital	54	63.0
Specialized	30	35.2
Others	1	1.9
**Departments**		
Surgery	23	42.6
Medicine	8	14.8
Burn &Plastic	13	24.1
Intensive care/emergency	3	5.7
Nursing supervisor/instructor/matron	3	5.7
Others	4	7.6

It was observed that only 16.7% of the respondents received training on burn management other than EMSB. Among the further burn training, the organizing institute included Dhaka Medical College, Mirpur Acid Survival Foundation and Sir Salimullah Medical College. Overall, 55.6% of the nurses had exposure to managing burn patients in their working place, and the rest did not need to do so. Those who managed burn patients, 56.7% manage every day. About 51.9% of respondents stated that they had the opportunity to use the knowledge and skill, acquired from EMSB training in managing burn patients, and only 3.7% did not think that it was valuable; the rest (44.4%) did not respond to the question. Those who had the opportunity to use the knowledge of EMSB among them huge majority (92.8%) mentioned that it helped manage severe burn patients (Table 2).

**Table 2:** Distribution of the respondents by burn training exposure and patient management.

Name of the variable	Frequency	Percentage
**Received other burn training (n = 54)**		
Yes	9	16.7%
No	45	83.3%
**Name of Institution of burn training (n = 9)**		
DMC	6	66.7%
Mirpur Acid Survival Foundation	1	11.1%
SSMC	2	22.2%
**Manage burn patient in working place (n = 54)**		
Yes	30	55.6%
No	24	44.4%
**Frequency of management of burn patient (n = 30)**		
Everyday	17	56.7%
At least twice a week	2	6.7%
1-3 times per month	6	20.0%
Rarely (less than once per month)	5	16.6%
**Opportunity to use the knowledge of EMSB (n = 54)**		
Yes	28	51.9%
No	2	3.7%
Don't know/Don't response	24	44.4%
**EMSB helpful for managing severe burn patient (n = 28)**		
Yes	26	92.8%
No	1	3.6%
Don't know/don't response	1	3.6%

**Figure 1:**
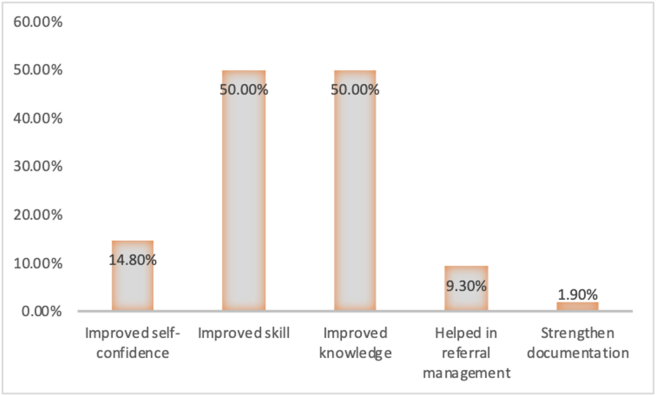
Distribution of the respondents according to their view on professional development after the EMSB course.

EMSB helped nurses in various ways to manage severe burn patients. About 14.8% reported that it helped them improve their confidence, 50.0% reported that it helped them improve their skill and knowledge. A small percentage (9.3%) mentioned it helped them in the referral system, and only one respondent thought the course helped to strengthen documentation (Figure 1).

**Figure 2:**
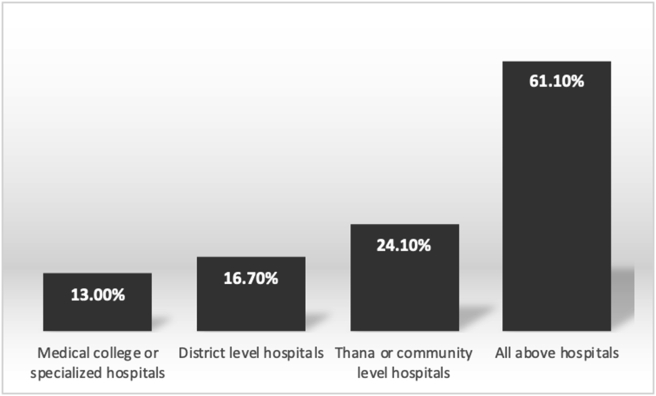
Level of the health care requiring EMSB according to respondents.

**Figure 3:**
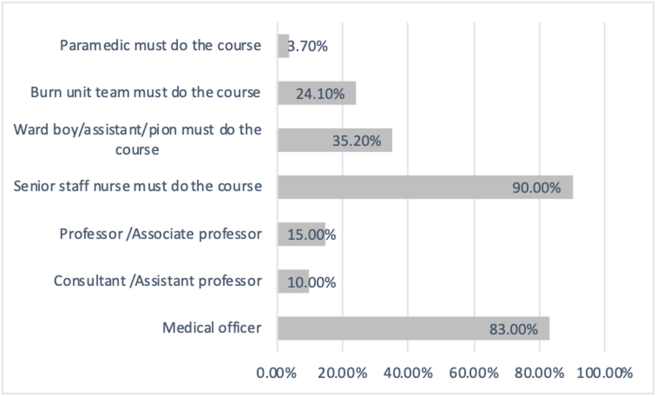
Health Care providers requiring EMSB according to respondents.

According to 61.1% of nurses, EMSB course should be availed by medical college hospitals or specialized hospitals, district-level hospitals and thana or community level hospitals (Figure 2). About 83% of respondents said that the medical officer should do the course. And ninety per cent of respondents mentioned that senior staff nurses should be trained in this course (Figure 3).

**Figure 4:**
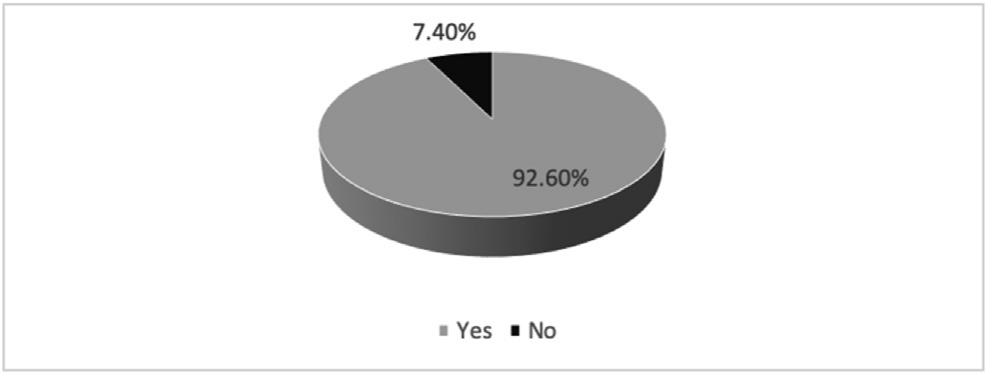
Necessity of EMSB refresher course according to respondents.

A majority (92.6%) of the respondents thought that refresher course on EMSB should be done (Figure 4).

Out of 54 Participants, 42 (77.8%) nurses mentioned challenges that they faced during the EMSB course. Among those short durations with vast theory and the practical session was mentioned by 35.2%, faced difficulty with understanding due to the English language by 20.4%, accommodation problem was observed by 3.7%. Based on the challenges, the 30 (55.7%) nurses suggested on the improvement of the EMSB course. Around 13% said the Bangla language needs to be reconsidered for training conduction for their convenience. Furthermore, the training time seemed to be an issue mentioned by 16.7% of the respondents (Table 3).

**Table 3:** Challenges of this course that mentioned by the respondents.

Name of the variables	Frequency (n = 54)	Percentage (%)
**Challenges faced by the respondents during this course**		
Short duration with vast theory and practical session	19	35.2
Language barrier	11	20.4
Accommodation problem	2	3.7
Honorarium not included	9	17
Training and test on the same day	1	1.8
Don't know/don't response	12	22.2
**Suggestion by the respondents on the improvement of EMSB**		
Bangla language was preferable for a training class	7	13.0
Training time needs to be increased	9	16.7
Honorarium needs to be paid	5	9.3
Patient management needs more focus	3	5.6
Arrangement of accommodation	1	1.8
EMSB training method needs to be improved	2	3.7
Severe burn session-practical class	3	5.6
Don't know/don't response	24	44.3

### 3.1 Focus group discussion findings

Four broader aspects of the EMSB course were discussed: the lecture session, skill stations, interactive discussions and moulage, written examination, course manual, and content. Faculty members responded based on the EMSB courses for the nurses in Bangladesh.

a. Lecture sessions

Faculty members mentioned that nurses easily followed the lecture sessions, understood that is faculty members are delivering. As those nurses are working closely with burn patients, they captured the theory. However, they were not interactive during the lectures, as the nurses were not fluent in speaking English.

One the faculty member mentioned-

*‘If the lecture copies could provide them earlier, it could be an option for them to better understand interaction.’*

b. Skill stations, interactive discussion and moulage

The faculty members stated that the nurses were proactive in different skills station, which was the most practical and hands-on skills learning session. However, in the interactive discussions, the nurses were not interactive due to their language barrier. However, they did very well in the burn case scenario-based discussion.

*“In the simulation, all nurses were well described on a simulated patient and all of them*

*qualified in the moulage examination, they did not face trouble to respond in English” – One of the faculty members described.”*

c. Written examination

Faculty members shared that the most challenging part was the written examination for the nurses. Most of the nurses faced a significant challenge to understand the meaning of the questions due to the English language. Although they knew the answer, they could not be able to respond, and in each of the nurse's courses, were, only 10–15% of the nurses could pass.

One of the faculty members spoke-

*“This course has been designed for doctors and nurses around the world. Nurses*

*in Bangladesh, during their graduation not practised English, therefore*

*it has become a challenging environment for them to understand the*

*meaning of questions and answers currently. If the questions are*

*provided in both English and Bengali, they might do far better.”*

d. Course manual and content

The faculty members described that nurses in Bangladesh might face a challenge to understand the course manual. Nurses received the manual 15 days before the course; however, they may not capture the language.

One of the faculty members who was the course director of EMSB at Australia New Zealand Burn Association (ANZBA) shared-

*“In Indonesia, they convert the course in their language, so that can be adapted in our country also. Bilingual manual and bilingual slides can be adapted in Bangladesh to make the course comfortable for both the doctors and nurses. Discussion and evaluation written test could be in Bangla.”*

Another faculty member added -

*“70–80% of portions can easily be understandable for all in English. The major problem is answering the questions in English. If Bangla questions were used for evaluation of the participants, that would drastically change the whole thing”.*

One of the key instructors mentioned.

*“A customized EMSB manual and content can be developed for the nurses in*

*Bangladesh, which includes course manual and content is developed in both*

*Bangla and English, besides that some pre-set mock questions can be given*

*in the manual for giving them scope to practice and understand the question style.”*

All faculty members agreed that language is the most significant barrier for the Bangladeshi graduated nurses to complete the EMSB course successfully. Although all nurses passed the moulage, they could not pass written in most cases. The faculty members also spoke that during the nurse's course, foreign participants from Nepal and India those who participated in Bangladesh all completed with a perfect score. Given that example, faculties recommended to ANZBA to work on the language of the course content, which will allow the Bangladeshi nurses to participate in the EMSB course and be qualified.

## 4 Discussion

EMSB course has been provided to the doctors and nurses for improvement of management on a burn patient. This study was conducted to evaluate nurses EMSB provider course in Bangladesh. Here we could not observe how this course helped them; manage burn patients, but only interviewed the respondents after they received the training based on their experiences; we found various aspects of this course that can help us improve it further. Of all the participants, 51.9% used the knowledge of EMSB, while the rest did not get an opportunity. The nurses also mentioned that medical officers, consultants, assistant professors, associate professors, surgeons, indoor medical officers, OT nurses, senior staff nurses, paramedics, burn unit team. Even non-medical general people should have trained in this course. Hence, selection criteria need more focused attention while inviting health care providers for the course. A UK study showed that paramedic and non-medical research scientists passed the course. So while choosing candidates, more emphasis needs to be given on the service delivery end rather than the sole background of the course participant [12]. Also, it can be considered to provide this training for military personnel and firefighters or any personnel who manages primary burn cases [14]. Also, 92.6% of the respondents mentioned having refreshers training, which was also suggested in literature from the Netherlands. Moreover, the study also indicated that refreshers training could be a short web-based for convenience [15].

Based on the self-evaluation of respondents after the course, it is seen that their confidence, skills and knowledge have improved but strengthening documentation and help in referral system management was mentioned by less than 10% of respondents; hence the course might need to emphasize these two areas for development. Earlier study on EMSB evaluation in Bangladesh had shown a large number of EMSB trained physicians able to support to manage emergency burn patients efficiently because of their development of skills through learning EMSB [16].

The challenges and suggestions reflect that EMSB being a training held only in Dhaka city is not favourable for nurses from around Bangladesh regarding accommodation. Though the training itself is free of cost, there is an added cost of commute and accommodation. Decentralizing the training sessions might be an option for overcoming this issue, or budgeting for the accommodation of nurses outside of Dhaka can be an option. The other challenging part is language; nurses in Bangladesh rarely have a stronghold of the English language, making it hard for them to following the lectures and manual; hence, the training might be reconsidered to deliver in the Bengali language along with the translated manual.

EMSB course for the nurse was a requirement to include in Bangladesh, particularly for those who are working in the peripherial healthcare facilities [16]. The EMSB creates its demand, and nurses are now interested in improving their burn management skills through this course and thought that other health professionals involved with burn management should be trained. A nationwide scale-up is desirable for addressing the overall burden of burn-related injuries.

### 4.1 Limitations of the study

The number of nurses’ EMSB courses was only three. In each course, 24 nurses were participants. Therefore we had to restrict our current study with those 72 nurse-participants only. The interviews were conducted over the telephone as the nurses’ duty stations were at different places around the country. In-person interview was not possible due to time and resource constraints. Nurses were interviewed at their workplaces over the phone. Therefore, considering huge work-pressure and limited time provisions, telephone interviews were planned to be in short. We were not able to collect alround information due to these practical limitations. We were able to do one focus group discussion with the key faculty members at, including the faculty from Interplast Australia and New Zealand, Australia & New Zealand Burn Association (ANZBA) as their faculty members visited Bangladesh only once during three of those courses. Therefore, representation of the qualitative data may not be generalized. However, one-third of the Bangladesh faculty members joined the FGD. Therefore the findings from the study have immense significance and wide implications in the field for near future for the healthcare policy makers and trainers.

## Declarations

### Author contribution statement

Animesh Biswas, Saidur Rahman Mashreky: Conceived and designed the experiments; Performed the experiments; Contributed reagents, materials, analysis tools or data; Wrote the paper.

Koustuv Dalal: Conceived and designed the experiments; Analyzed and interpreted the data; Contributed reagents, materials, analysis tools or data; Wrote the paper.

Rifat Ara Sawon, Cinderella Akbar Mayaboti: Analyzed and interpreted the data; Wrote the paper.

### Funding statement

This research did not receive any specific grant from funding agencies in the public, commercial, or not-for-profit sectors.

### Data availability statement

The authors do not have permission to share data.

### Declaration of interests statement

The authors declare no conflict of interest.

### Additional information

No additional information is available for this paper.
